# The impact of an innovative web-based school nutrition intervention to increase fruits and vegetables and milk and alternatives in adolescents: a clustered randomized trial

**DOI:** 10.1186/s12966-017-0595-7

**Published:** 2017-10-16

**Authors:** Karine Chamberland, Marina Sanchez, Shirin Panahi, Véronique Provencher, Jocelyn Gagnon, Vicky Drapeau

**Affiliations:** 1 Département de l’éducation Physique, Faculté des Sciences de l’éducation, Université Laval, Québec City, QC G1V 0A6 Canada; 2 Centre de recherche de l’Institut Universitaire de Cardiologie et de Pneumologie de Québec, Québec City, QC Canada; 3 Institut sur la Nutrition et les Aliments Fonctionnels, Université Laval, Québec City, QC Canada; 4 Département de Kinésiologie, Université Laval, Québec City, QC G1V 0A6 Canada; 5 Centre de Recherche Interuniversitaire sur la Formation et la Profession Enseignante (CRIFPE-Laval), Université Laval, Québec City, QC Canada

**Keywords:** Adolescents, School-based nutrition intervention, Vegetables and fruit, Milk and alternatives, Dairy products, Web-based platform, Eating behaviours

## Abstract

**Background:**

The increase in overweight and obesity in adolescents and its health-related consequences highlight the need to develop strategies, which could help them adopt healthy eating habits. The objective of this study was to evaluate the impact of an innovative web-based school nutrition intervention (Team Nutriathlon) aimed at promoting the consumption of vegetables and fruit (V/F) and milk and alternatives (M/A) in high school students and to identify facilitators and/or barriers influencing its success.

**Methods:**

Ten classes of first and second year secondary students (grades 7 and 8) from the Québec City region were randomized into two groups (control *n* = 89 and intervention *n* = 193). Participants in the intervention (Team Nutriathlon) were to increase their consumption of V/F and M/A using an innovative web-based platform, developed for this study, over 6 weeks. The control group followed the regular school curriculum. The number of servings of V/F and M/A consumed by students per day was compared between the two groups before, during, immediately after and 10 weeks after the intervention using a web-based platform. Main outcome measures included V/F and M/A servings and facilitators and/or barriers of program success. Repeated measures linear fixed effects models were used to assess the impact of Team Nutriathlon on V/F and M/A consumption. A *P*-value of <0.05 was considered significant.

**Results:**

Students in the intervention reported a significant increase of 3 servings and 1.8 servings per day of V/F and M/A, respectively, compared to the control group (*P* < 0.05); however, this was only observed in the short-term. Some factors contributing to the success of Team Nutriathlon included the team aspect of the program, use of the technology and recording results outside of classroom hours.

**Conclusion:**

Team Nutriathlon represents an innovative web-based nutrition program which positively impacts V/F and M/A consumption among high school students. Using web-based or technological platforms may help youth adopt healthy eating habits that will have implications later in adulthood; however, further studies are needed to determine their long-term effects.

**Trial registration:**

NCT03117374 (retrospectively registered).

## Background

The prevalence of obesity has doubled in the last 30 years and affects more than one in three children and adolescents in Canada [1]. Obesity has also been associated with increased physical and psychosocial problems that may have repercussions later in life [2, 3]. Adolescence is recognized as a critical period characterized by rapid and significant growth with high energy requirements [4]. During this period adolescents learn to adopt healthy lifestyles that play a key role in their current and future health [5]. Although adolescence is synonymous with the development of autonomy, the environment remains an important influence on lifestyle habits [5]. This may partly explain why the dietary habits of adolescents are suboptimal. Among the food groups, a lack of consumption of vegetables and fruit (V/F) and milk products (M/A) and alternatives (such as enriched soy beverages) may lead to suboptimal dietary intakes. According to a Québec survey on the health of youth, more than 50% of adolescents do not consume the recommended Canada Food Guide servings for V/F and M/A [6]. Considering that youth attend school regularly and continuously, and that V/F and M/A consumption is not optimal in this population, establishing initiatives targeting V/F and M/A habits in the youth school environment has great potential for success.

In addition to possessing high nutrient density, V/F and M/A may play an important role in childhood obesity treatment and prevention. In a previous study, we observed that among 41 food groups, an increase in whole fruit and low-fat milk intake appeared to be the two specific groups consistently associated with better weight control; however, this was observed in adults [7]. Acute studies have demonstrated that the incorporation of V/F into children’s meals can decrease energy intake by lowering energy density [8]. One intervention also showed a beneficial effect of increasing V/F on children’s eating habits compared to a more restrictive intervention that included a reduction in fat [9]. This is line with studies showing that an increase in V/F or dairy products often results in a concomitant decrease in unhealthy snacks/foods [10–13]. Furthermore, inverse associations have been observed between consumption of dairy products and body weight in children [14]. Thus, a high consumption of these two food groups appears to represent a useful strategy to prevent or treat obesity.

Several studies confirm that nutrition intervention initiatives in schools offer a promising approach for reaching adolescents and influencing changes in their eating behaviours [15–20]. More specifically, previous studies highlight that most efficient intervention to improve VF in children/adolescents are based on a variety of approaches, including nutrition education, increased availability of healthy foods, free or subsidized food programs, environmental changes, and parental involvement [16, 18, 21]. Although these studies have shown an increase in the consumption of V/F, few have focused on increasing the variety of these foods, including the consumption of M/A and engage youth in a specific behavioural change process.

In line with these observations, our research team recently evaluated the impact of a nutrition program called “Team Nutriathlon” where the objective was to improve the consumption and diversification of V/F and M/A in primary school children by helping them to develop autonomy in the management of a healthy diet [22]. The implementation of the first version of the program resulted in a significant increase in the consumption of V/F and M/A in children suggesting a role for nutrition programs in schools. Although this study used paper and pencil to record, methods utilizing internet and game technologies have been increasingly used approaches designed to promote health behaviour changes. Accordingly, school-based intervention programs which incorporate technology (e.g. internet use) in obesity prevention in youth have been suggested to influence eating behaviours and increase the consumption of V/F and M/A, particularly when integrated into the school environment [23–27]. However, none of these interventions have included team challenges which support peer influence and motivation as well as a web-based component combined with a face-to-face regulation process that supports the development of autonomy in food choices and may lead to sustained behavioural changes. Therefore, the objective of the present study was to (1) assess the impact of a web-based version of Team Nutriathlon, an innovative web-based nutrition intervention, on the consumption of V/F and M/A and (2) identify facilitators and/or barriers influencing its success among high school students. Since adolescents have been raised with the evolution of technology and are more receptive to using new tools [28], it was hypothesized that the web-based version of Team Nutriathlon would be a practical way to increase the consumption V/F and M/A in this population.

## Methods

### Participants and recruitment

This randomized, clustered intervention was conducted between 2011 and 2013 and included 10 classes of grades secondary I and II (grades 7 and 8) from three different high schools in Québec City, Canada. These classes were randomized into an intervention (*n* = 6) or control group (*n* = 4). The number of classes and students per class varied from one school to another. The selected schools were of medium to high socioeconomic levels according to the Deprivation Index of the Ministry of Education of Québec.

Schools were recruited on a voluntary basis via email invitation and sent across the region of Québec. Teachers interested in participating and whose environment matched the inclusion criteria of the study (i.e. be a school consisting of at least two secondary I or II classes corresponding to 13 and 14 year-old youth) were invited to contact the leaders of the study. Classes were randomized to either the intervention (i.e. Team Nutriathlon) or control (i.e. regular school curriculum) group. To better control for different school environments and seasonal influences on eating habits, both groups were randomized in the same school and during the same season. Because of the efficacy of the intervention in our previous study and to increase the motivation of teachers to participate in the current study, more participants were included in the current intervention. Only data for which parents and students gave their consent and assent, respectively, were used for the compilation and analysis of the results. This study was approved by the Ethics Committees for Research in Psychology and Educational Sciences at Université Laval.

### Team Nutriathlon intervention

The current study examined the impact of an innovative web-based version of Team Nutriathlon aimed at improving the quality of each participant’s diet by increasing and diversifying their consumption of V/F and M/A. The initial Team Nutriathlon “paper and pencil” version was designed as an 8-week school-based nutrition intervention and team challenge [22]. This version was shortened over a 6-week period and adapted for secondary school students (13–14 years old) and for compatibility with the characteristics of the school environment to provide remote management via an interactive web interface.

#### Web-based team Nutriathlon platform

The web-based platform was secured by an information technology specialist who developed a password secured website to allow online participation by recording the daily consumed portions of V/F and M/A. Thus, in order to participate in Team Nutriathlon, each participant was provided with a USERID and password to login. The website provided a six-week calendar where each participant was asked to record, twice a day, their consumption of V/F and M/A from Monday to Friday. Specifically, each day on the calendar included two icons (an apple and a milk carton) representing V/F and M/A, respectively. By clicking on each of these icons, participants were able to add their servings of the various foods at each meal (breakfast, morning snack, lunch, afternoon snack, dinner, and evening snack) which were divided further into various categories. Thus, V/F and M/A were separated into colors based on their nutritional density: (1) green (vegetables rich in folic acid such as broccoli); (2) orange (V/F rich in beta-carotene such as carrots); (3) purple (vegetables rich in potassium and folic acid such as red cabbage); (4) yellow (fruits rich in vitamin C such as oranges); (5) red (fruits rich in potassium and vitamin C such as cranberries); and (6) blue (M/A rich in calcium and vitamin D including milk, cheese and yogurt). The web-based Team Nutriathlon platform automatically calculated the means of V/F and M/A consumed by each participant. It also allowed the presentation of “summary reports” which presented the team totals for the quantity and variety of V/F and M/A that were recorded and compared these results with the Team Nutriathlon goals. These summary reports were essential for the “regulation periods” that were held every 2 weeks. The web-based platform also indicated the individual and team symbolic prizes that were attributed to participants at the end of the program.

Although Team Nutriathlon is not a theory-based intervention, it includes key behaviour change techniques related to self-determination models. These include goal settings, providing feedback, identifying barriers and solutions, reinforcement and social support [29] which can positively influence one’s extrinsic and intrinisic motivation, a strong determinant of the adoption of healthy eating habits. Team Nutriathlon was also aimed at promoting the development of the students’ autonomy which means to promote their decision-making and control over their behaviors relating to their food choices. Development of autonomy fostered by the Team Nutriathlon is one important element of the self-determination theory [30] and has been associated with the adoption of healthy eating behaviors [31]. The regulation periods aimed to improve student’s autonomy allowed them to self-manage their progress toward individual and team objectives. More specifically, it provided time for students to reflect on their recent V/F and M/A consumption, to analyze their progress and to find individual- and team-based strategies to help them increase their servings in the following weeks. In this context, Team Nutriathlon, although not a theory-based intervention, appears to promote key components of the self-determination theory such as competence and autonomy [30].

Since the program was geared towards the development of autonomy, students decided when to use the platform; however, teachers also invited them to access two 5-min periods during the day to record their information. Furthermore, although parents were not directly involved, materials such as pamphlets describing the details of Nutriathlon and its goals were shared with them. Participants were also able to request purchasing V/F and M/A from the various food categories from their parents.

#### Implementation phases of team Nutriathlon

The implementation of Team Nutriathlon consisted of three distinct phases including the preparatory, implementation and balance sheet phases. During the preparatory phase, the program co-ordinator provided training and instruction to teachers on the implementation and execution of the web-based program modalities. In line with the competence of “adopting a healthy and active lifestyle” from the Québec Education Program [32], they also learned how to help Nutriathlon participants apply various strategies to reach Team Nutriathlon individual and team goals. A program implementation guide was provided to each teacher to help identify tasks related to the program.

During the six-week implementation phase, students were encouraged to increase their consumption of V/F and M/A in order to satisfy the individual and team requirements of Team Nutriathlon. In the individual component, students were challenged to reach an average daily consumption of the recommended servings of V/F (6 servings/day) and M/A (3 servings/day) based on Canada’s Food Guide by age group [33]. In the team component, comprising of three to five students, success depended on two important factors including the “quantity” and “variety” of the foods eaten. For the “quantity” team goal, team members must have accumulated, after 6 weeks, a total number of portions of V/F and M/A, which varied by number of participants in the team. To reach the “variety” team goal, this consumption must have been equally distributed into the six categories of foods associated with colors. The aim of the “variety” goal was to increase the likelihood that students would taste new foods during the program. The individual goals required students to attain a specific “quantity” of servings and team goals were based on both “quantity” of intake and “variety”. Follow-ups, also known as “regulation periods”, were planned after the second, fourth and 6 weeks of Team Nutriathlon in order to specifically develop the autonomy of youth regarding their food choices. Regulation periods are based on an analysis of summary reports provided by the website. The summary report includes two-week cumulative intakes of V/F and M/A servings for each student and team (week 2 = mean of weeks 1 and 2; week 4 = mean of weeks 3 and 4, etc.) and indicates the students and teams that attained the goals. During the regulation periods, students analyzed their consumption over the previous 2 weeks, the effectiveness of their strategies and planned new strategies that enabled them to adjust their consumption to better meet the requirements of Team Nutriathlon with the help of their teachers.

Finally, during the last regulation period (seventh week), a final phase was organized by the project managers in order to determine the final results of the 6-week changes in the consumption of V/F and M/A of students and to provide a balance sheet. During this phase, individuals and teams were provided with symbolic prizes on the website and were characterized into “Goûteur” (taster) for reaching the individual goals based on the “quantity” and into “Gastronome” (1st place); “Gourmet” (2nd place) “Dégustateur” (3rd place) for reaching team goals based on the “quantity” or “variety” of foods consumed.

#### Control group

The control group was not exposed to the Nutriathlon challenge objectives (i.e. no team) or Nutriathlon website access. They were only exposed to the usual school curriculum which did not include a specific nutrition program. Throughout the study, participants had access to a plain website in order to record their consumption of V/F and M/A at specific periods. It should be noted that the data collection on the web-based platform for the control group in no way referred to Team Nutriathlon. In addition, to reduce the emphasis on V/F, M/A, eating behaviours and body weight, the research objective that was presented to the participants was “to explore the effects of different lifestyle interventions on health and well-being”.

### Data collection

#### Quantitative data

Quantitative data was collected to compare the consumption of V/F and M/A among students participating in Team Nutriathlon and the control group. Each group underwent an initial evaluation period (week 0, pre-) where baseline consumption of V/F and M/A using a plain web-based platform was obtained without referring to Team Nutriathlon in any way. With the assistance of a study co-ordinator, teachers explained to students the concept of servings and modalities on how to use the web-based platform. Anthropometric measurements were taken during the first week of data collection. Participants were weighed using a bioimpedance balance and height was measured using a stadiometer by the study co-ordinator. Overweight/obesity was determined using BMI z-scores according to the criteria of the World Health Organization [34].

**Fig. 1:**
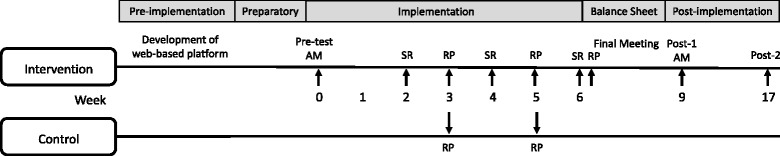
Research protocol (AM: Anthropometric measures; RP: Regulation Periods; SR: Summary Reports). Consumption of V/F and M/A were collected for all participants at pre-test, weeks 3 and 5, Post-1 and Post-2. Anthropometric measures were taken at pre-test and Post-1

Following this initial evaluation, the experimental group participated in Team Nutriathlon while the control group was exposed to the usual school curriculum program. Consumption of V/F and M/A during the intervention was collected using the Team Nutriathlon web-based platform daily during a six-week period (Monday to Friday) for the intervention and at two different times for the control group on the plain web-based platform (weeks 3 and 5, Monday to Friday). Consumption of V/F and M/A after Team Nutriathlon (week 9, post-1) and 10 weeks after the end of the program (week 17, post-2) was also obtained on a plain web-based platform. It is important to note that the web-based platform used for collecting pre- and post-consumption data of V/F and M/A for both groups and during the study for the control group was similar to the Team Nutriathlon web-based platform, but did not include any information or references on Team Nutriathlon procedures. Anthropometric measurements were also taken at the end of Team Nutriathlon (post-1). Finally, although the intervention was 6 weeks, the post-1 assessment period was postponed until week 9 due to a 1 week spring break. The research protocol is shown in Fig. 1.

#### Qualitative data

The semi-structured group interview method was conducted among a subgroup of students to document the barriers and facilitators related to program success. Qualitative data were collected using a grid adapted to the interview with students. This grid contained 12 open-ended questions on five different themes: barriers and facilitators to the success of Team Nutriathlon, motivation, perception of results, appreciation and future benefits (Table 1). All interviews were conducted only in the intervention group. Among the intervention groups, four Nutriathlon teams were included and selected on the basis of their Team Nutriathlon performance (i.e. the two teams who performed the best and two teams who performed poorly). Their performance was evaluated based on their final summary reports. These interviews were conducted with the teams (four to five students per interview) by one research assistant trained in the analysis of qualitative studies. In these interviews, barriers and facilitators related to the success of the program were discussed with open-ended questions.

**Table 1:** Interview questions for students containing 12 open-ended questions on five different themes

Theme 1: Barriers and Facilitators	
Question 1	In your opinion, what facilitated (people, environment, personal skills, etc.) your success in Nutriathlon? How did these things help you? What did your teacher to do to facilitate success?
Question 2	In your opinion, what inhibited (people, environment, personal skills, etc.) your success in Nutriathlon? How did these things keep you from successfully achieving your goal? How did you manage to overcome these obstacles? How did your teacher help you overcome these obstacles?
Theme 2: Motivation	
Question 3	What influenced you to get involved in Nutriathlon?
Question 4	What prevented you from getting involved in Nutriathlon?
Question 5	How did you motivate yourself? What did your teacher do to motivate you?
Theme 3: Perception of Results	
Question 6	Did you learn something in the area of physical activity/nutrition?
Question 7	Are there tips/strategies that you will remember in the future to help you do physical activity and/or consume V/F and M/A?
Theme 4: Appreciation	
Question 8	What did you like in Nutriathlon? (e.g. activities, time and effort required, level of difficulty, teammates, etc.)
Question 9	What did you not like in Nutriathlon? (e.g. activities, time and effort required, level of difficulty, teammates, etc.)
Question 10	Would you want to redo Nutriathlon if you had the chance? Why?
Theme 5: The Future	
Question 11	Following Nutriathlon, do you think it is important to do physical activity and/or eat V/F and M/A?
Question 12	Following Nutriathlon, do you feel like doing physical activity and/or eating V/F and M/A? If so, do you think you will be able to?

### Statistical analyses

Statistical analyses were performed using JMP analysis software (JMP 7.0, SAS Institute, Cary, NC, USA). The sample size was calculated based on our previous studies to detect significant differences in consumption of V/F of 0.5 servings per day with an alpha level of 0.01 and a power (1-β error probability) of 0.90. For the quantitative data analysis, the primary variable was the daily servings of V/F and M/A consumed as reported by youth on the web-based platform. The repeated measures linear fixed effects models were used to assess the impact of Team Nutriathlon (independent variable) on the consumption of V/F and M/A (dependent variable). These analyses took the clustering randomization effect into account using the fixed effects model (i.e. random effect for classes). Tukey-Kramer’s post hoc test was performed to determine between-group differences at each time. In the analysis, results with greater than 15 portions of V/F (*n* = 23% female, 96% intervention) and 10 servings of M/A (*n* = 9% female, 100% intervention) were excluded since their values were greater than twice the standard deviation. Body weight and BMI z-scores were analyzed using the Student’s t-test for paired samples to assess the weight status of students before and after Team Nutriathlon. The data were reported by means ± standard deviation and a *P*-value of <0.05 was considered significant.

Analysis of the interviews was done using the manual content analysis method and the open-content analysis model [35]. According to L’Ecuyer (1990), three steps are essential to achieving a content analysis: a) preliminary reading and establishment of a list of statements; b) choice and definition of the classification units; and c) a categorization process. Verbatim transcripts of each of the four interviews were therefore necessary. The content analysis resulted in six categories: Nutriathlon logistics, student’s Team Nutriathlon perceptions, factors that facilitate or inhibit the success of Nutriathlon, impact of Team Nutriathlon on students and their families, student engagement during Team Nutriathlon and student’s future involvement in the program. Analysis of the information collected from students was carried out by a research professional qualified in qualitative analysis methods. To ensure scientific validity, another research professional also qualified in qualitative analyses external to the project also repeated the analyses in order to confirm the categories resulting from the content analysis. Subsequently, the two professionals met to discuss the results and form a consensus.

## Results

**Fig. 2:**
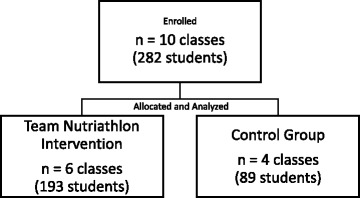
Total number of classes and participants in the intervention and control groups

A total of 282 students (61% female) participated in the study with 193 students (62% female) in the intervention and 89 students (58% female) in the control group (Figure 2). Initially, 42 students (18%) were overweight (48% female, 67% intervention) and 25 (11%) students were obese (64% female, 68% intervention). No significant differences were observed between groups at baseline (pre-test, Table 2). Moreover, body weight change (post-1-baseline) was not significantly different between the intervention and control groups (1.3 ± 1.9 kg vs 1.1 ± 1.9 kg, *P* = 0.37, respectively). Similar results were observed for changes in BMI z-scores (−0.09 ± 0.02 kg for the intervention group and −0.04 ± 0.03 kg for the control group, *P* = 0.12).

**Table 2:** Baseline participant characteristics

	Group	
	Intervention (n = 193)	Control (n = 89)
Age	13.7 ± 0.9	13.4 ± 0.8
Weight (kg)	54.0 ± 12.5	52.7 ± 12.9
BMI z-scores^a^	0.4 ± 0.1	0.4 ± 0.1
Female (%)	62	58
Male (%)	38	42
Overweight (%)^b^	17	19
Obese (%)^b^	10	11

^a^38 students (53% female; 71% intervention) had missing data for weight. Analyses of data related to BMI are based on information from 244 students (62% female, 68% intervention)

^b^45 students (53% female, 69% intervention) had missing data for weight (*n* = 38) or age (*n* = 7). Analyses of data related to overweight and obesity percentages are based on information from 237 students (62% female, 68% intervention)

### Impact of team Nutriathlon on the consumption of V/F and M/a

**Fig. 3:**
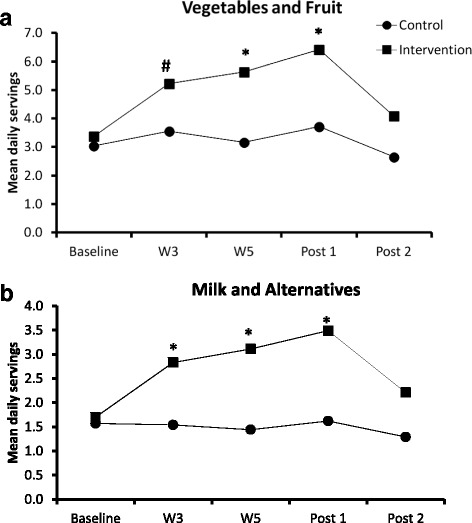
A comparison of intervention (Team Nutriathlon) and control groups on the consumption of (**a**) V/F and (**b**) M/A for the different data collection periods (W = week). *Significant differences between the intervention and control groups (V/F: W5 *P* = 0.0002; Post-1 *P* = 0.002 and M/A: W3 *P* = 0.0005; W5 *P* < 0.0001; Post-1 *P* < 0.0001, respectively). ^#^Trend for a difference between the intervention and control groups (V/F: W3 *P* = 0.08)

At baseline (pre-test), there were no significant differences between the control and intervention groups on the consumption of V/F and M/A (Figure 3). An effect of time, group and an interaction (time x condition) was observed for the consumption of V/F (F = 17.8, *P* < 0.0001, F = 12.6, *P* = 0.01 and 6.6. *P* < 0.0001, respectively) and M/A (F = 12.80, *P* < 0.0001, F = 22.99, *P* = 0.002 and 9.8, *P* < 0.0001, respectively). At weeks three and five and at the end of Nutriathlon (post-1), the intervention group reported a higher consumption of V/F and M/A compared to the control group (Figure 3). In contrast, 10 weeks after the end of the intervention (post-2), there were no significant differences in the consumption of V/F (*P* = 0.64) and M/A (*P* = 0.40) between the intervention and control groups. Moreover, there was no effect of sex on the consumption of V/F and M/A (*P* < 0.48; data not shown).

The consumption of V/F between the pre-test and post-1 was increased to 0.39 servings for the control and 3.4 for the intervention group. Students in the intervention group consumed on average 6.4 daily servings of V/F while the control group consumed an average of 3.7 servings at the end of the intervention (post-1).

Milk and alternative consumption between the pre-test and post-1 was increased to 0.14 servings in the control and 1.9 servings in the intervention group. Students in the intervention group consumed an average of 3.5 daily servings while the control group consumed an average of 1.6 servings per day after the intervention (post-1).

### Facilitators and barriers to success in team Nutriathlon perceived by students

During interviews, students identified factors facilitating the success of the program (Table 3). Because Nutriathlon promotes teamwork, it has been identified as a good strategy to encourage program participation. For the majority of students, the involvement of teachers proved to be a facilitating factor because they reminded students to record the consumed servings of V/F and M/A on the web-based platform. Moreover, to encourage students to participate in Team Nutriathlon, teachers helped students achieve their goals by providing tips to increase their consumption of V/F and M/A. Some strategies provided to students during the “regulation periods” also came from the research team, other students and/or parents. Several students mentioned the importance of parental input during the program. Moreover, parents were involved in different ways with the adolescents by encouraging them to eat more V/F and M/A, reminding them to note their results on the website, increasing the amount of V/F during meals at home and in lunch boxes and buying more healthy foods at the grocery store.

**Table 3:** Facilitators and barriers contributing to the success of Team Nutriathlon according to students

Facilitators	Barriers
• Entering data on a web-based platform • Team aspect of the program • Involving teachers • Obtaining strategies • Family environment	• Lack of attendance for recording data on the website • Technological aspects (dysfunction of the user code and incompatibility with other technological tools)

As for barriers to the success of Team Nutriathlon two major themes emerged: one related to “attendance” and the other to the “technological aspect”. Attendance at school for entering data on the website was identified as one important factor influencing success of the program. Some students experienced difficulty in attendance for completing their compilation sheets on the web-based platform. Several students struggled to take responsibility outside of school hours to record their data. Barriers related with the “technological aspect” included problems with personal code entry which provided access to the Team Nutriathlon website and incompatibility of the web-based platform with some devices such as the tablet.

## Discussion

This study aimed to evaluate the impact of an innovative web-based school nutrition intervention (i.e. Team Nutriathlon) on the consumption of V/F and M/A in high school students, a population where there is a need to improve eating habits and develop nutrition programs. The results of this study suggest that Team Nutriathlon is a promising strategy that promotes the consumption of V/F and M/A in this population, at least in the short-term.

Quantitative results confirmed the positive impact of Team Nutriathlon among youth by the significant increase observed in the consumption of V/F and M/A in the intervention group at weeks 3, 5 and 9 (post-1) even though consumption returned to initial levels 10 weeks after the intervention (post-2). Similarly, several studies have documented the effectiveness of school-based interventions in increasing the consumption of V/F in youth in the short-term [15–20]. In a systematic review on school-based interventions aimed at promoting V/F intake among children and adolescents, 10 out of 15 studies demonstrated an increase in V/F consumed from 0.3 to 1 serving/day for the intervention groups [18]. However, Team Nutriathlon observed an additional average 2.7 servings of V/F consumed per day which represents a 53% increase in V/F consumption between pre- and post-1 for the intervention group suggesting a significant impact of the web-based version of this program. Of note, the majority of the previously cited studies are interventions focusing on nutrition education and environmental change while the emphasis of Team Nutriathlon is on the modification of behaviour and development of skills, which may also explain the positive results observed.

As indicated earlier, the effectiveness of school-based interventions targeting M/A consumption is less documented than V/F. The short-term effectiveness of an intervention that encouraged consumption of a specific number of M/A servings in children compared to a control group [36] and the inclusion of nutrition education interventions in the school curriculum has been suggested to be beneficial in increasing M/A [37]. Studies on interventions encouraging M/A consumption in a healthy eating environment have achieved significant results and represent an increase by one serving a day which is similar to our results (two servings more for the intervention group following Team Nutriathlon) [38].

Team Nutriathlon participants identified various factors facilitating the success of the program. Students suggested that the use of technology (i.e. web-based platform) in the framework of this program facilitated its implementation. A meta-analysis of interventions using computer technology in the form of computer games that increase motivation in youth was shown to be effective in increasing the consumption of V/F [21] and may be an effective way to encourage students to change and adopt behavior that may be beneficial to their health [39]. These results are not surprising considering that youth learn more by using a fun platform. The majority of students who participated in Team Nutriathlon found it simple and appreciated being “part of a team”. Moreover, teachers and peer support also contributed to the success of Team Nutriathlon. Several studies have found that social dynamics are a key element to the adoption of healthy lifestyles [40–42] and the involvement of teachers and peers encourage the consumption of V/F and M/A in youth [43]. The family environment was another facilitating factor important for students and has been shown to positively impact eating habits [44–47]. Parents, through their influence on food accessibility [48, 49], support and being a role model [49, 50] may directly influence the consumption of certain foods. Throughout the duration of Team Nutriathlon, students were introduced to strategies that enabled them to more easily achieve their goals. In our study, students reported that the strategies provided by teachers, parents, researchers and those discovered by themselves were factors facilitating the consumption of V/F and M/A. Developing strategies or “practical skills” (e.g. adding more V/F in the lunch box) and obtaining support from teachers and parents would allow students to increase their V/F consumption [51].

Students also identified some barriers to the success of Team Nutriathlon. First, students did not find it convenient to use the platform to enter their consumption of V/F and M/A outside of school hours. This may have been due to a lack of motivation or because students were not in the school environment. Second, students identified technical difficulties with the use of the user code and incompatibility of the platform with certain technological tools such as tablets. However, these latter factors remained out of the control of students and should be modified and considered further in the continuation of this research and other web-based intervention studies.

Although the long-term impact of this type of intervention on the health and behavior of young adolescents is difficult to assess, our study shed some light on the short-term beneficial effects of a program that promotes the adoption of healthy eating habits. Some factors may partly explain the impact of Team Nutriathlon in the longer term. Participation in a team was a factor facilitating engagement in the program and peer and/or teacher support were important elements in increasing the consumption of V/F and M/A. Thus, the little access to the support of peers and teachers after completion of the program may have contributed to the decrease in consumption 10 weeks after. A second factor was the lack of follow-up with participants after the intervention. Frequent contact with the participants has been suggested to ensure success of a program [52]. Since the daily monitoring and control periods offered by teachers and the research team had ended after Team Nutriathlon, students found themselves in a less structured environment and it was more difficult for them to continue. Finally, it is possible that the duration of Team Nutriathlon was not long enough to change behavior. Because of the high-school agenda, Team Nutriathlon was implemented over a 6-week period, which is less than the original 8-week period that was found to be effective in younger children in the short and longer terms (10 weeks post) [22]. Since the proportion of different weight categories of the participants in this study was consistent with Canadian population statistics, weight issues less likely to explain the results of the study. More research is needed to establish a significant link between the decrease in consumption of V/F and M/A 10 weeks after the end of Team Nutriathlon and the various factors discussed.

This study has several strengths. First, the large mixed sample increases the generalizability of the results. The inclusion of a mixed methodology (quantitative and qualitative measurements) highlights both the impact of Team Nutriathlon on V/F and M/A consumption and student perceptions. This study was therefore able to identify the facilitating factors and barriers perceived by students to adopt healthy habits as part of a school nutrition intervention. Accordingly, it is mainly an extracurricular program, which means that it takes minimal class time to implement (about 4 h for a 6-week period). It also requires minimal human resources such as one coordinator to enter the team’s name and give a password to each student and a teacher to introduce the Team Nutriathlon at the beginning and supervise the “regulation periods”. Even though the Team Nutriathlon is not a multi-level intervention (i.e. targets not only the individual, but also the environment), it could well be integrated into a school setting that has implemented a nutrition education program in the curriculum or increased the availability of V/F and M/A in their environment. It is also important to highlight that Team Nutriathlon focuses on modifying a single behaviour (i.e. eating habits such as V/F and M/A consumption). Although this has not been extensively studied, this approach has been suggested to be more effective compared to interventions that focus on changing multiple behaviours [53]. Finally, the innovative aspect of this intervention stems from the combination of a team challenge and a web-based component combined with face-to-face interactions that include regulation periods, with the goal of developing autonomy in food choices.

This study is not without some limitations. The program was carried out in schools where it is difficult to analyze the long-term results due to school holidays, changes in the cohort of students every year and departure of some students. Second, the results are not generalizable to all high school students, particularly those with low socio-economic statuses, since the study was conducted in a population of high school students from relatively high socio-economic backgrounds. Third, participation in Team Nutriathlon on a voluntary basis may lead to possible bias already present related to the motivation of students and teachers of the schools involved in the project. Although the level of motivation was not measured in this study, it is clear that this factor could influence the success of the Nutriathlon. This has been highlighted in Team Pentathlon, the physical activity version of the Nutriathlon program [54]. A higher level of motivation among teachers participating in this study could be due to different factors such a better knowledge of nutrition and health and/or personal beliefs. Future studies may include implementation of training programs in schools aimed to increase the level of motivation or influence beliefs related to these behaviours. Fourth, as the servings of V/F and M/A consumed were self-reported by students, social desirability may have influenced their consumption. Students may be encouraged to include more portions of V/F and M/A than actually consumed, because they are aware that this is what is best for their health; however, it is difficult to control this bias. Fifth, due to budgetary constraints and the design of the study, this project did not include process evaluation which is essential for capturing what is delivered and how it is carried out in practice, in comparison to the planned theoretical intervention. This process also allows one to know which adaptations have been made in order to fit the intervention into different contexts and determine its impact on the outcomes. However, in the context of the present study, a step-by-step guide was provided to each teacher to help them standardize the implementation of Nutriathlon and thus, increase intervention fidelity. Moreover, information on participant compliance was available. Students were encouraged to access the platform 80% of the time (4 out of 5 days). This information was available to teachers throughout the intervention period allowing them to encourage students that were less using the platform (less than 80% of the time). Finally, because dietary intakes were not assessed using 24-h food recalls or direct observations, it was not possible to document if V/F and M/A changed the overall diet quality. Nevertheless, because no differences in z-BMI scores were observed, it may be hypothesized that the increase in V/F and M/A did not result in an increase in total energy intake over the course of the study. One possible explanation is that the increase in V/F and M/A may have helped to decrease the consumption of unhealthy snacks. This is supported by our previous study demonstrating that the implementation of Team Nutriathlon in primary schools result in an increase in V/F and M/A at snack time [22], and by other results showing that the introduction of a V\F program in schools is associated with a decrease in the consumption of unhealthy snacks [10, 11, 13]. Together, these results indicate that Team Nutriathlon increases the consumption of V/F and M/A more specifically between meals which may help to decrease the consumption of less healthy foods.

## Conclusion

In conclusion, this study indicates that Team Nutriathlon is an innovative web-based nutrition program which positively impacts V/F and M/A consumption among high school students, at least in the short term. Web-based technologies favouring the development of autonomy in food choice seems thus to represent an interesting avenue to promote healthy eating in adolescents. Although educational institutions are a good choice for implementing web-based intervention in youth, it may be relevant to implement Team Nutriathlon in other types of environments popular among youth including summer camps or daycare. Furthermore, using web-based or technological platforms may help youth adopt healthy eating habits that will have implications later in adulthood; however, further studies are needed to determine their long-term effects.

## Abbreviations

M/A: Milk and alternatives
V/F: Vegetables and fruit

## References

[1] Statistics Canada (2015). Body mass index of children and youth, 2012 to 2013.

[2] Daniels SR (2009). Complications of obesity in children and adolescents. Int J Obes.

[3] Lambert M, Delvin EE, Levy E, O'Loughlin J, Paradis G, Barnett T, McGrath JJ (2008). Prevalence of cardiometabolic risk factors by weight status in a population-based sample of Quebec children and adolescents. Can J Cardiol.

[4] Health Canada. Do Canadian adolescents meet their nutrient requirements through food intake alone? 2012 [cited 2016 August 20]; Available from: http://www.hc-sc.gc.ca/fn-an/surveill/nutrition/commun/art-nutr-adol-eng.php.

[5] Institut national de santé publique du Québec. Sociocultural, environment and lifestyle of adolescents: Better understanding for better action. 2014 [cited 2016 August 15]; Available from: https://www.inspq.qc.ca/pdf/publications/1774_EnvSocioCultHabVieAdos.pdf.

[6] Institute of Statistics Quebec. Québec Health Survey of High School Students (QHSHSS) 2010–11. 2012 [cited 2016 August 15]; Available from: http://www.stat.gouv.qc.ca/enquetes/sante/eqsjs_an.html.

[7] Drapeau V, Despres JP, Bouchard C, Allard L, Fournier G, Leblanc C, Tremblay A (2004). Modifications in food-group consumption are related to long-term body-weight changes. Am J Clin Nutr.

[8] Pourshahidi LK, Kerr MA, McCaffrey TA, Livingstone MB (2014). Influencing and modifying children's energy intake: the role of portion size and energy density. Proc Nutr Soc.

[9] Epstein LH, Gordy CC, Raynor HA, Beddome M, Kilanowski CK, Paluch R (2001). Increasing fruit and vegetable intake and decreasing fat and sugar intake in families at risk for childhood obesity. Obes Res.

[10] Tak NI, Te Velde SJ, Singh AS, Brug J (2010). The effects of a fruit and vegetable promotion intervention on unhealthy snacks during mid-morning school breaks: results of the Dutch Schoolgruiten project. J Hum Nutr Diet.

[11] Overby NC, Klepp KI, Bere E (2012). Introduction of a school fruit program is associated with reduced frequency of consumption of unhealthy snacks. Am J Clin Nutr.

[12] Bere E, te Velde SJ, Smastuen MC, Twisk J, Klepp KI (2015). One year of free school fruit in Norway--7 years of follow-up. Int J Behav Nutr Phys Act.

[13] Andersen LB, Arnberg K, Trolle E, Michaelsen KF, Bro R, Pipper CB, Molgaard C (2016). The effects of water and dairy drinks on dietary patterns in overweight adolescents. Int J Food Sci Nutr.

[14] Lu L, Xun P, Wan Y, He K, Cai W (2016). Long-term association between dairy consumption and risk of childhood obesity: a systematic review and meta-analysis of prospective cohort studies. Eur J Clin Nutr.

[15] Cohen JF, Kraak VI, Choumenkovitch SF, Hyatt RR, Economos CD (2014). The CHANGE study: a healthy-lifestyles intervention to improve rural children's diet quality. J Acad Nutr Diet.

[16] Evans CE, Christian MS, Cleghorn CL, Greenwood DC, Cade JE (2012). Systematic review and meta-analysis of school-based interventions to improve daily fruit and vegetable intake in children aged 5 to 12 y. Am J Clin Nutr.

[17] Howerton MW, Bell BS, Dodd KW, Berrigan D, Stolzenberg-Solomon R, Nebeling L (2007). School-based nutrition programs produced a moderate increase in fruit and vegetable consumption: meta and pooling analyses from 7 studies. J Nutr Educ Behav.

[18] Knai C, Pomerleau J, Lock K, McKee M (2006). Getting children to eat more fruit and vegetables: a systematic review. Prev Med.

[19] Reynolds KD, Franklin FA, Binkley D, Raczynski JM, Harrington KF, Kirk KA, Person S (2000). Increasing the fruit and vegetable consumption of fourth-graders: results from the high 5 project. Prev Med.

[20] Siega-Riz AM, El Ghormli L, Mobley C, Gillis B, Stadler D, Hartstein J, Volpe SL, Virus A, Bridgman J (2011). The effects of the HEALTHY study intervention on middle school student dietary intakes. Int J Behav Nutr Phys Act.

[21] Delgado-Noguera M, Tort S, Martinez-Zapata MJ, Bonfill X (2011). Primary school interventions to promote fruit and vegetable consumption: a systematic review and meta-analysis. Prev Med.

[22] Drapeau V, Savard M, Gallant A, Nadeau L, Gagnon J (2016). The effectiveness of a school-based nutrition intervention on Children's fruit, vegetables, and dairy product intake. J Sch Health.

[23] Hamel LM, Robbins LB (2013). Computer- and web-based interventions to promote healthy eating among children and adolescents: a systematic review. J Adv Nurs.

[24] Di Noia J, Contento IR, Prochaska JO (2008). Computer-mediated intervention tailored on transtheoretical model stages and processes of change increases fruit and vegetable consumption among urban African-American adolescents. Am J Health Promot.

[25] Mauriello LM, Ciavatta MM, Paiva AL, Sherman KJ, Castle PH, Johnson JL, Prochaska JM (2010). Results of a multi-media multiple behavior obesity prevention program for adolescents. Prev Med.

[26] Winett R, Roodman A, Winett S, Bajzek W, Rovniak L, Whiteley J (1999). The effects of the Eat4Life internet-based health behavior program on the nutrition and activity practices of high school girls. J Gend Cult Health.

[27] Randi Schoenfeld E, Ng P, Henderson K, Wu SY (2010). Using the internet to educate adolescents about osteoporosis: application of a tailored web-education system. Health Promot Pract.

[28] Burstein GR, Lowry R, Klein JD, Santelli JS (2003). Missed opportunities for sexually transmitted diseases, human immunodeficiency virus, and pregnancy prevention services during adolescent health supervision visits. Pediatrics.

[29] Michie S, Richardson M, Johnston M, Abraham C, Francis J, Hardeman W, Eccles MP, Cane J, Wood CE (2013). The behavior change technique taxonomy (v1) of 93 hierarchically clustered techniques: building an international consensus for the reporting of behavior change interventions. Ann Behav Med.

[30] Ryan RM, Patrick H, Deci EL, Williams GC (2008). Facilitating health behaviour change and its maintenance: interventions based on self-determination theory. Eur Health Psychologist.

[31] Pelletier LG, Dion SC, Slovinec-D'Angelo M, Reid R (2004). Why do you regulate what you eat? Relationships between forms of regulation, eating behaviors, sustained dietary behavior change, and psychological adjustment. Motiv Emot.

[32] Ministère de l'Éducation du Québec, Programme de formation de l'école québécoise (Quebec Education Training Program). 2001.

[33] Health Canada. Eating Well with Canada's Food Guide. 2011 [cited 2016 August 30]; Available from: http://www.hc-sc.gc.ca/fn-an/food-guide-aliment/index-eng.php.

[34] World Health Organization. Facts and Figures on Childhood Obesity. 2014; Available from: http://www.who.int/end-childhood-obesity/facts/en/.

[35] L'Écuyer R, Méthodologie de l'analyse développementale de contenu (Method of developmental content analysis) Méthode GPS et concept de soi (GPS method and self-concept). 1990, Québec: Presses de l’Université du Québec. 490.

[36] Albala C, Ebbeling CB, Cifuentes M, Lera L, Bustos N, Ludwig DS (2008). Effects of replacing the habitual consumption of sugar-sweetened beverages with milk in Chilean children. Am J Clin Nutr.

[37] Nicklas TA (2003). Calcium intake trends and health consequences from childhood through adulthood. J Am Coll Nutr.

[38] Abreu S, Santos R, Moreira C, Santos PC, Vale S, Soares-Miranda L, Autran R, Mota J, Moreira P (2014). Relationship of milk intake and physical activity to abdominal obesity among adolescents. Pediatr Obes.

[39] Casazza K, Ciccazzo M (2007). The method of delivery of nutrition and physical activity information may play a role in eliciting behavior changes in adolescents. Eat Behav.

[40] Teyhen DS, Aldag M, Centola D, Edinborough E, Ghannadian JD, Haught A, Jackson T, Kinn J, Kunkler KJ, Levine B, Martindale VE, Neal D, Snyder LB, Styn MA, Thorndike F, Trabosh V, Parramore DJ (2014). Key enablers to facilitate healthy behavior change: workshop summary. J Orthop Sports Phys Ther.

[41] Baggio A, Kispal L, Woodruff S. Associations between Encouraging Peers or Receiving Teacher/Principal Encouragement and Fruit/vegetable Intake among Students in Grades 5-8 in Northern Ontario, Canada. Edinburgh: The International Society for Behavioral Nutrition and Physical Activity Abstract Book June 3-6 2015.

[42] Mcdonald D. Evaluating the Effectiveness of a Youth Peer-education Intervention for Increasing Physical Activity and Consumption of Fresh Fruits and Vegetables. Edinburgh: The International Society for Behavioral Nutrition and Physical Activity Abstract Book June 3-6 2015.

[43] Agence de la santé publique du Canada. La santé des jeunes Canadiens: un accent sur la santé mentale. 2012; Available from: http://www.phac-aspc.gc.ca/hp-ps/dca-dea/publications/hbsc-mental-mentale/school-ecole-fra.php. Accessed Jan 2017.

[44] Dietz WH, Gortmaker SL (2001). Preventing obesity in children and adolescents. Annu Rev Public Health.

[45] Pearson N, Biddle SJ, Gorely T (2009). Family correlates of fruit and vegetable consumption in children and adolescents: a systematic review. Public Health Nutr.

[46] Rasmussen M, Krolner R, Klepp KI, Lytle L, Brug J, Bere E, Due P (2006). Determinants of fruit and vegetable consumption among children and adolescents: a review of the literature. Part I: quantitative studies. Int J Behav Nutr Phys Act.

[47] Van Der Horst K, Oenema A, Ferreira I, Wendel-Vos W, Giskes K, van Lenthe F, Brug JA (2007). Systematic review of environmental correlates of obesity-related dietary behaviors in youth. Health Educ Res.

[48] Olson BH, Chung KR, Reckase M, Schoemer S (2009). Parental influences on dairy intake in children, and their role in child calcium-fortified food use. J Nutr Educ Behav.

[49] Sharma SV, Hoelscher DM, Kelder SH, Diamond P, Day RS, Hergenroeder A (2010). Psychosocial factors influencing calcium intake and bone quality in middle school girls. J Am Diet Assoc.

[50] Gillman MW, Rifas-Shiman SL, Frazier AL, Rockett HR, Camargo CA, Field AE, Berkey CS, Colditz GA (2000). Family dinner and diet quality among older children and adolescents. Arch Fam Med.

[51] Henderson M, Gray-Donald K, Rabasa-Lhoret R, Bastard JP, Barnett TA, Benedetti A, Chaput JP, Tremblay A, Lambert M (2014). Insulin secretion and its association with physical activity, fitness and screen time in children. Obesity (Silver Spring).

[52] Hendrie GA, Brindal E, Baird D, Gardner C (2013). Improving children's dairy food and calcium intake: can intervention work? A systematic review of the literature. Public Health Nutr.

[53] Sweet SN, Fortier MS (2010). Improving physical activity and dietary behaviours with single or multiple health behaviour interventions? A synthesis of meta-analyses and reviews. Int J Environ Res Public Health.

[54] Michaud V, Nadeau L, Martel D, Gagnon J, Godbout P (2012). The effect of team pentathlon on ten- to eleven-year-old childrens’ engagement in physical activity. Phys Educ Sport Pedagog.

